# Predictive factors of Quality of Life in older adults during the COVID-19 pandemic

**DOI:** 10.1186/s40359-022-00882-w

**Published:** 2022-07-17

**Authors:** Hossein Khorani, Fatemeh Mohammadi, Zahra Hosseinkhani, Seyedeh Ameneh Motalebi

**Affiliations:** 1 Student Research Committee, Qazvin University of Medical Sciences, Qazvin, Iran; 2 Social Determinants of Health Research Center, Research Institute for Prevention of Non-Communicable Diseases, Qazvin University of Medical Sciences, Qazvin, Iran; 3 Metabolic Diseases Research Center, Research Institute for Prevention of Non-Communicable Diseases, Qazvin University of Medical Sciences, Qazvin, Iran

**Keywords:** Aged, Quality of life, COVID-19, Fear

## Abstract

**Background:**

Given the vulnerability of older people to COVID-19, it is important to consider their physical and mental wellbeing and quality of life (QoL) in the COVID-19 pandemic. Therefore, the present study was aimed to identify the QoL and its predictive factors among a sample of Iranian older adults during the COVID-19 pandemic.

**Methods:**

This descriptive and cross-sectional study was conducted on 500 older people residing in Qazvin, Iran, from May 22th to November 21rd, 2021. Multistage cluster sampling method was used for selecting the eligible older adults. Data were collected using the demographic checklist, fear of COVID-19 scale, and Elderly Quality of Life Questionnaire (LIPAD). The multivariate regression model was used for determining the predictive factors of QoL in older people.

**Results:**

The mean age of older participants was 69.17 ± 6.75 years old. The results of multivariate regression model showed that fear of COVID-19, age, marital status, level of education, living arrangement, and economic situation were the significant predictors of QoL in the older adults during the COVID-19 pandemic.

**Conclusions:**

It is recommended to pay close attention to divorced, lonely, and illiterate older people and those with low economic situation during the COVID-19 pandemic.

## Background

China reported the emergence of a new influenza-like virus that infected many people in Wuhan in December 2019 [1]. Despite efforts to control the virus across the city, the disease has spread rapidly in China and other countries worldwide [2]. The virus was very similar to the coronavirus that appeared in China from 2002 to 2003, and it was known as a severe acute respiratory syndrome (SARS). For this reason, the World Health Organization (WHO) named the virus SARS COVID-2 and the resulting disease COVID-19 in February 2020 [3]. COVID-19 is a warning shot for the world [4, 5]. According to the WHO report, in June 2021, three million Iranian patients infected with COVID-19 have been identified, of which more than 83,000 deaths were recorded [6].

Older adults are more susceptible to COVID-19 infection, caused by a defective immune system response to infectious diseases [7]. In this regard, Liu et al. showed that the prevalence of COVID-19-related deaths was higher among people 60 years and older than in other population groups [8]. U.S. Centers for Disease Control and Prevention also reported that although people aged 65 years and older make up 17% of the total population, 31% of people infected with the virus, 45% of inpatients, 53% of ICU inpatients, and 80% of COVID-19-related deaths occur among the older adults [9]. Older adults are more likely to get severe illnesses and death from COVID-19 [10]. Factors that can cause poor health outcomes in older adults include age-related physiological changes, chronic diseases such as cardiopulmonary disease, diabetes, dementia, and concomitant medicine use [11]. Older adults are the first to be quarantined due to their physical conditions and greater vulnerability to COVID-19 disease [12]. During the pandemic, long-term social distancing can increase unnecessary fear, stress, and worries about the future [13]. Similarly, Ferreira et al. reported a higher level of anxiety and lower QoL during home quarantine of COVID-19 [14]. QoL is a continuous multifaceted concept that refers to physical, social, functional, and psychological well-being [15]. Previous studies have reported increased mental disorders such as fear, anxiety, and depression during the COVID-19 pandemic. In a recent study conducted in China (2020) on 1556 older people, 37.1% suffered from depression and anxiety [16]. Furthermore, Negarestani et al. reported that more than three-quarters of the older adults did not have good mental health during the COVID-19 pandemic [17].

One common feature of infectious disease compared with other conditions is fear. Fear is directly related to its transmission, morbidity, and mortality rates. Older people are also faced with psychological stress resulting in fear and anxiety [18] due to the high transmission and mortality rate of the disease, social isolation, economic problems, and difficulties in reaching health services [19, 20]. On the other hand, spreading COVID-19 and misinformation can increase public fear and affect the QoL [12]. Correspondingly, Nguyen et al. reported that older adults were more likely to have higher depression and a more inadequate QoL than their younger counterparts [21]. This fear further resulted in other psychosocial issues, including stigmatization, discrimination, and loss [22].

To the best of our knowledge, few studies have been recently conducted on the QoL of older adults and its related factors during the COVID-19 pandemic. Considering the high prevalence of COVID-19 among the older adults, as a vulnerable age group, and the importance of improving their QoL, the present study aimed to determine the predictive factors of QoL among a sample of Iranian older adults during the COVID-19 pandemic.

## Methods

This descriptive and cross-sectional study was performed on 500 older adults aged 60 years and over residing in Qazvin, Iran. The multistage cluster sampling method was used for selecting older people. First, Qazvin city was divided into five regions, including North, South, East, West, and Center based on the city map. Then, the researchers identified the locations where the older people usually gather including mosques, parks, and daycare centers in each region. Ten parks and twenty masques were randomly selected from these five geographic areas. Furthermore, the samples were selected from two daily older care centers with members from all five regions. Then questionnaires were distributed among those who attended in the selected locations. During the data collection, 43 older people refused to participate. The analysis was conducted on 500 older adults at the end and the responding rate was 92.1%. Inclusion criteria included age 60 years old and over and the willingness to participate in the study. The older people who suffered from mental or cognitive conditions and severe physical illnesses that inhibit effective communication were excluded from the study.

Taking into account, type I error rate = 0.01, test power = 0.95, and the correlation coefficient between fear of COVID-19 and QoL (r = − 0.188) [23], the sufficient sample size based on the following formula was determined at 500.

$$n=\frac{{{(\mathrm{Z}}_{1-\frac{\alpha }{2}}+{Z}_{1-\beta } )}^{2}}{{w}^{2}}+3$$

### Instruments

A demographic information checklist, fear of COVID-19 scale [18], and Elderly Quality of Life Questionnaire (LIPAD) [24] were used for collecting the data.

Demographic information checklist includes age, gender, marital status, number of children, level of education, job, economic situation, living arrangement, insurance status, history of infection or death of family members, friends, or neighbors due to COVID-19 as well as the number of physical illnesses.

Fear of COVID-19 Scale: This scale was developed by Ahorsu et al. [18] to assess the level of fear of COVID-19 among the Iranian population in 2020. The answers are scored based on a five-point Likert ranging from strongly disagree to strongly agree. The possible score range is 7 to 35 with a higher score indicating greater fear of COVID-19. Internal consistency and test–retest reliability of this scale was reported to be 0.82 and 0.72, respectively. In this study, the value of Cronbach alpha was calculated to be 0.965 (95% confidence interval (CI) 0.960–0.970) for all seven items.

LIPAD: LIPAD was designed by De Leo et al. [24], and it was used in three cities of Leiden in the Netherlands, Padua in Italy, and Helsinki in Finland. This questionnaire consists of 31 questions that measure seven dimensions of QoL including physical dimensions (5 questions), self-care (6 questions), depression and anxiety (4 questions), cognitive (5 questions), social (3 questions), life satisfaction (6 questions), and sexual issues (2 questions). A higher score means a better QoL. This questionnaire is scored on a four-point Likert scale ranging from 0 (worst case) to 3 (best case). The possible score range is also 0 and 93. Hesamzadeh et al. calculated the validity and reliability of this questionnaire among Iranian older adults and reported that its Cronbach alpha reliability coefficient is 0.831 [25]. In the present study, the value of Cronbach alpha was calculated at 0.953 (95% CI 0.946–0.958) for all 31 items.

Questionnaires were completed through face-to-face interviews by the first author from May 22th to November 21rd, 2021.

### Data analysis

The data were analyzed by the Statistical Package for the Social Sciences, version 20.0 (SPSS Inc., Chicago, Illinois, USA). Quantitative variables were described using means and standard deviations (SD) and qualitative variables by frequencies and percentages. To determine the predictors of QoL, first, univariate regression was run and then the variables which were significantly associated with QoL (*p* ≤ 0.2) [26] were entered in the multivariate regression model.

QQ-plot and box-plot confirmed that there was a normal distribution of the QoL data. The skewness (− 0.579) and the kurtosis (− 0.430) values were also in the acceptable ranges. As Byrne [27] stated that if the skewness value is between − 2 to + 2 and the kurtosis value is between − 7 to + 7, normality of the data could be assumed. To assess multicollinearity issues, the variance inflation factor (VIF) of QoL was computed (1.01) which was in the acceptable range. The homoscedasticity was also evaluated and confirmed.

## Results

In the present study, the mean (SD) of the participants’ age was 69.17 (6.75) years and the possible age range was 60 to 89 years. The demographic characteristics of the study participants are presented in Table 1.

**Table 1:** Demographic characteristics of the study participants

Demographic characteristics		Numbers	Percentage
Gender	Female	236	47.2
	Male	264	52.8
Marital status	Single	29	5.8
	Married	271	54.2
	Divorced	51	10.2
	Widowed	149	29.8
Level of educational attainment	Illiterate	72	14.4
	Elementary	160	32.0
	Secondary	218	43.6
	Academic	50	10.0
Economic situation	Low	51	10.2
	Average	251	50.2
	Good	159	31.8
	Excellent	39	7.8
Insurance	No	75	15.0
	Yes	425	85.0
Job	Housewife/Unemployed	184	36.8
	Retired	263	52.6
	Employed	53	10.6
Living arrangement	With spouse	188	37.6
	With spouse and children	75	15.0
	With children	82	16.4
	Alone	146	29.2
	Others	9	1.8
Number of children	0	35	7.0
	1–3	107	21.4
	4–5	131	26.2
	> 5	227	45.4
COVID-19 infection in family, friends, or neighbors	No	126	25.2
	Yes	374	74.8
COVID-19 death in family, friends, or neighbors	No	331	66.2
	Yes	169	33.8
Number of physical disease	0	45	9.0
	1–2	226	45.2
	≥ 3	229	45.8

Variable	Mean	SD	
Age (years)	69.17	6.75	

According to the results, the mean (SD) score of fear of COVID-19 was 21.05 (8.92) and the mean (SD) of QoL was 59.31 (14.80).

The results of multivariate regression model showed that fear of COVID-19, age, marital status, level of education, living arrangement, and economic situation were the most significant predictors of QoL in the older adults during the COVID-19 pandemic. QoL had a negative association with age (β = − 0.49, *p* < 0.001) and fear of COVID-19 (β = − 0.44, *p* < 0.001). Participants had higher QoL, if they had secondary (β = 4.45, *p* = 0.008) or academic (β: 6.25, *p* = 0.10) educational level, average (β = 9.18, *p* < 0.001), and good (β = 12.68, *p* < 0.001) or excellent (β = 14.64, *p* < 0.001) economic situations. Those who lived alone (β = − 8.44, *p* = 0.009) or with others (β = − 13.28, *p* = 0.005) reported lower QoL compared with those lived with a spouse. Furthermore, divorced older adults reported lower QoL(β = − 5.79, *p* = 0.029) compared with those who were single (Table 2).

**Table 2:** Predictors for QOL among older adults during COVID-19 pandemic

Variables		Crud β coefficient (CI 95%)	Adjusted β coefficient (CI 95%)
Fear		− 0.74(− 0.87, − 0.61) *p* < 0.001	− 0.44(− 0.57, − 0.32) *p* < 0.001
Age		− 0.90(− 1.08, − 0.73) *p* < 0.001	− 0.49(− 0.66, − 0.32) *p* < 0.001
Gender		− 3.70(− 6.28, − 1.11) *p* = 0.005	0.97(− 1.37, 3.31) *p* = 0.416
Marital status	Single	1	1
	Married	1.77(− 3.26, 6.79) *p* = 490	− 4.23(− 11.26, 2.79) *p* = 0.237
	Divorced	− 10.77(− 16.75, − 4.79) *p* < 0.001	− 5.792(− 10.99, − 0.58) *p* = 0.029
	Widow	− 13.18(− 18.39, − 7.95) *p* < 0.001	− 2.46(− 7.62, 2.69) *p* = 0.349
Level of education	Illiterate	1	1
	Elementary	4.48(0.68, 8.28) *p* = 0.021	2.67(− 0.46, 5.80) *p* = 0.095
	Secondary	11.52(7.88, 15.16) *p* < 0.001	4.45(1.15, 7.74) *p* = 0.008
	Academic	20.74(15.81, 25.67) *p* < 0.001	6.25(1.49, 11.01) *p* = 0.010
Economic situation	Low	1	1
	Average	10.50(6.51, 14.49) *p* < 0.001	9.18(5.89, 12.47) *p* < 0.001
	Good	18.68(14.50, 22.86) *p* < 0.001	12.68(9.00, 16.35) *p* < 0.001
	Excellent	26.64(21.12, 32.18) *p* < 0.001	14.64(9.69, 19.60) *p* < 0.001
Job	Housewife-unemployed	1	1
	Retired	9.10(6.45, 11.75) *p* < 0.001	0.99(− 2.18, 4.16) *p* = 0.540
	Employed	12.90(8.60, 17.19) *p* < 0.001	1.23(− 10.33, 12.80) *p* = 0.843
Living arrangement	With spouse	1	1
	With children and spouse	− 0.04(− 3.59, 3.5) *p* = 0.982	0.87(− 2.01, 3.77) *p* = 0.552
	With children	− 12.86(− 16.31, − 9.42) *p* < 0.001	− 5.64(− 12.45, 1.15) *p* = 0.104
	Alone	− 13.85(− 16.73, − 10.98) *p* < 0.001	− 8.44(− 14.75, − 2.14) *p* = 0.009
	Others	− 11.57(− 20.45, − 2.69) *p* = 0.011	− 13.28(− 22.43, − 4.12) *p* = 0.005
Insurance	Yes	8.96(5.40, 12.52) *p* < 0.001	− 0.13(− 3.34, 3.08) *p* = 0.938
COVID in family	Yes	− 6.63(− 9.58, − 3.70) *p* < 0.001	− 0.12(− 3.34, 3.08) *p* = 0.938
COVID death in family	Yes	− 7.03(− 9.71, − 4.34) *p* < 0.001	− 0.72(− 1.49, 0.04) *p* = 0.065
Physical disease	Yes	− 8.05(− 12.37, − 3.73) *p* < 0.001	− 2.49(− 5.82, 0.83) *p* = 0.141

β: Standard regression coefficient, t: Test statistics, QoL: Quality of Life, SD: Standard Deviation, CI: Confidence Interval

## Discussion

The present study aimed to determine QoL predictors in a sample of Iranian older adults during the COVID-19 pandemic. The results of the current study showed a high and negative association between age and QoL, which is consistent with the results of national [28] and international studies [29–31]. In this regard, it can be stated that the high prevalence of chronic physical and mental diseases and the decline in physical activity can be practical factors in reducing the QoL of older adults [32]. However, Brown and Roose reported that age was not associated with QoL [33]. This discrepancy may be attributed to socio-cultural differences, the type of instrument used, or the average age of the study participants.

Fear of COVID-19 was another predictor of QoL in older adults during the pandemic. In this regard, Fahad et al. showed that people who experienced a history of fear, anxiety, depression, and stress reported lower QoL during the pandemic [34]. Hosseini-Moghaddam et al. also reported that fear of COVID-19 infection, death anxiety, and misinformation on social media could increase depression and reduce the QoL among older adults [35]. Furthermore, Korsi Dorene et al. showed that the perceived fear of COVID-19 infection was negatively associated with QoL [36]. In other words, fear of COVID-19 infection is associated with an increased feeling of insecurity [37], fear of losing loved ones, increasing psychological distress, and a decrease in QoL [36, 37]. Bluma et al. also reported that older adults at higher risk for COVID-19 infection, and approximately, 37% of them experienced depression and anxiety, had a better QoL than other age groups [38]. This discrepancy could be attributed to the cultural differences, the target study population, and the QoL level before the COVID-19 outbreak.

The present study results showed that divorced older adults reported lower QoL compared with those were single. These findings are in accordance with Gutiérrez-Vega et al., which showed that single older adults reported higher QoL than divorced adults [39]. In fact, divorced people lose a feeling of belongingness and emotional support [40], which can negatively affect their QoL [41]. Divorced people can also experience higher pressure and stress than married or single people, as Conversano et al. reported that living with a spouse resulted in lower distress during the COVID-19 pandemic [42]. However, the impact of marital status on the QoL in different societies is partly influenced by their cultural and social conditions, as Seangpraw et al. [43] could not find this result among the rural older adults in Northern Thailand.

Education level was found to be a significant predictor of QoL. Specifically, older adults with higher education levels (secondary and academic education compared with illiterate individuals) were more likely to have higher QoL. This finding is consistent with the results of previous studies [44–47]. It may be that well-educated people, particularly those with academic education, are generally linked to better occupational prospects, higher income, and greater health literacy and health-related information that improves their QoL.

The present study results revealed that living alone is an influential factor in reducing the QoL of older adults during the COVID-19 pandemic. Living alone is a risk factor for emotional problems, such as loneliness and depression, decreasing the QoL [48–50]. Wu reported that social isolation and loneliness are the main risk factors associated with poor physical and mental health status among older adults in the context of COVID-19 [51]. Rumas et al. also found that during the pandemic, loneliness was associated with reduced in all domains of QoL [52]. Living alone, especially during the COVID-19 pandemic can lead to anxiety, fear, and distress among older adults. Furthermore, living alone can limit access to health care services among older adults during this pandemic.

Moreover, results showed that older adults with low economic status were more likely to report lower QoL. In this regard, El-Hage et al. stated that financial worries and employment during the COVID-19 pandemic could significantly reduce the QoL of individuals [53]. Ping et al. also reported that low income and economic status played an important role in reducing the health-related QoL of older adults by increasing health concerns during the pandemic [54].

### Limitations

The present study was performed on the community-dwelling older adults, so, it is difficult to generalize the results to the institutionalized older adults. Furthermore, due to the self-report method for filling out questionnaires that some seniors may not have given a real answer. By giving the necessary explanations about the objectives of the study, an attempt was made to reduce this limitation. Finally, only older adults who could be present in public places participated in this study, so the results may not generalize to older people with physical disabilities.

## Conclusion

In general, findings showed that several factors could impact the QoL of older adults. In this regard, age, marital status, fear of COVID-19, living alone, being illiterate, and low economic situation were found as predictive factors of QoL in this group of people. Therefore, considering demographic characteristics and providing a theoretical and practical approach for reducing the fear of COVID-19 is essential for improving the QoL of this vulnerable population.

## Data Availability

All data generated or analyzed during this study are included in this published article [supplementary file:SPSS file].

## Abbreviations

QoL: Quality of Life
WHO: World Health Organization
SARS: Severe Acute Respiratory Syndrome
US: United State
ICU: Intensive Care Unit
